# Hepatectomy risk assessment with functional magnetic resonance imaging (HEPARIM)

**DOI:** 10.1186/s12885-021-08830-4

**Published:** 2021-10-23

**Authors:** Mohamed Elsharif, Matthew Roche, Daniel Wilson, Susmita Basak, Ian Rowe, Dhakshina Vijayanand, Richard Feltbower, Darren Treanor, Lee Roberts, Ashley Guthrie, Raj Prasad, Mark S. Gilthorpe, Magdy Attia, Steven Sourbron

**Affiliations:** 1 Leeds Teaching Hospitals NHS Trust, St James University teaching Hospital, Level 6, Bexley Wing. St James’s Hospital, Beckett Street, Leeds, LS9 7TF England; 2 Biomedical Imaging Sciences Department, Leeds Institute of Cardiovascular and Metabolic Medicine LIGHT Laboratories, University of Leeds, Leeds, LS2 9JT England; 3 Leeds Institute for Data Analytics, School of Medicine, University of Leeds, Worsley Building, Clarendon Way, Leeds, LS2 9NL England; 4 Department of Clinical Pathology, and Department of Clinical and Experimental Medicine, Linköping University, Linköping, Sweden; 5 Center for Medical Image Science and Visualization (CMIV), Linköping University, Linköping, Sweden; 6 Level 4, Welcome Trust Brenner Building, St. James’s University Hospital, Leeds, LS9 7TF England; 7 Cardiovascular and Diabetes Research, Leeds Institute of Cardiovascular and Metabolic Medicine LIGHT Laboratories, University of Leeds, LS2 9JT Leeds, England; 8 IICD - Sheffield, Sheffield, England

**Keywords:** Post hepatectomy liver failure, Risk assessment, Functional MRI, Gadoxetate, Indocyanine green, Future liver remnant

## Abstract

**Background:**

Post hepatectomy liver failure (PHLF) remains a significant risk in patients undergoing curative liver resection for cancer, however currently available PHLF risk prediction investigations are not sufficiently accurate.

The Hepatectomy risk assessment with functional magnetic resonance imaging trial (HEPARIM) aims to establish if quantitative MRI biomarkers of liver function & perfusion can be used to more accurately predict PHLF risk and FLR function, measured against indocyanine green (ICG) liver function test.

**Methods:**

HEPARIM is an observational cohort study recruiting patients undergoing liver resection of 2 segments or more, prior to surgery patients will have both Dynamic Gadoxetate-enhanced (DGE) liver MRI and ICG testing.

Day one post op ICG testing is repeated and R15 compared to the Gadoxetate Clearance (GC) of the future liver remnant (FLR-GC) as measure by preoperative DGE- MRI which is the primary outcome, and preoperative ICG R15 compared to GC of whole liver (WL-GC) as a secondary outcome.

Data will be collected from medical records, biochemistry, pathology and radiology reports and used in a multi-variate analysis to the value of functional MRI and derive multivariant prediction models for future validation.

**Discussion:**

If successful, this test will potentially provide an efficient means to quantitatively assess FLR function and PHLF risk enabling surgeons to push boundaries of liver surgery further while maintaining safe practice and thereby offering chance of cure to patients who would previously been deemed inoperable. MRI has the added benefit of already being part of the routine diagnostic pathway and as such would have limited additional burden on patients time or cost to health care systems. (*Hepatectomy Risk Assessment With Functional Magnetic Resonance Imaging - Full Text View -* *ClinicalTrials.gov*, n.d.)

**Trial registration:**

ClinicalTrials.gov, ClinicalTrials.gov NCT04705194 - Registered 12th January 2021 – Retrospectively registered

**Supplementary Information:**

The online version contains supplementary material available at 10.1186/s12885-021-08830-4.

## Background

The liver is the most common site of metastases in patients with colorectal cancer (CRC). Up to two thirds of CRC patients go on to develop liver metastasis during the course of their disease and 18% actually presents with liver metastasis at their first consultation [1–3].. Primary liver cancers are the fifth most commonly occurring cancer in men and the ninth in women worldwide with 840,000 new cases diagnosed in 2018 alone [4]. This includes mainly two types of cancer: hepatocellular carcinomas (HCC), often associated with chronic liver disease and cholangiocarcinomas (CCA), tumours of the biliary tree that most commonly occur in the perihilar region of the liver, but also within the liver parenchyma and as a result require extensive resections.

Whilst several treatment modalities exist the only curative option for these patients is surgical resection ranging from minor parenchymal preserving metastasectomy to major liver resection removing up to 75% of the liver. The latter group is technically challenging and associated with significant risks and complications; potentially the most serious of these being post-hepatectomy liver failure or PHLF [5] which can ultimately result in death. PHLF occurs in 5–10% of patients undergoing major hepatectomy due to inability of the remnant liver to maintain its synthetic, excretory, and detoxifying functions and not regenerate at a sufficient pace. PHLF ranges in severity from transient hepatic insufficiency while the liver regenerates to full-blown fulminant hepatic failure [6–8]. The latter group is associated with a high risk of mortality of up to 54% [9].

Preoperative methods used to reduce such risk include portal vein embolisation (PVE) of the part of the liver to be removed in future surgery. PVE will result in diverting blood flow to the contralateral side and subsequent hypertrophy of the liver remnant (May and Madoff 2012). A second alternative is to perform the operation over two stages which often involves clearing of tumour(s) on one side and ligating the portal vein branch to other side at first stage followed by second stage after several weeks to remove the part of liver that has atrophied. Alternatively, a similar outcome can be achieved via an open surgical procedure called ALPPS (associating liver partition and portal vein ligation in staged hepatectomy). ALPPS involves two operations that are spaced in a shorter time interval. All of these techniques are associated with increased complications of different magnitude. PVE, the least invasive carries risk of some morbidity and failure to induce hypertrophy in a proportion of patients. The two stage operations have increased morbidity of two operations and ALPPS is associated with a significant increase in mortality. The risk is high enough for ALPPS with tumours like Choilangiocarcinoma at liver hilum that it is currently contraindicated for these tumours. The risk of failure to induce hypertrophy persists with two stage hepatectomy whereas ALPPS, despite the significantly increased complications has a higher chance of succeeding in inducing hypertrophy.

Selection of patients for surgery, precise planning of surgery and assessing the need for interventions to induce liver hypertrophy would benefit greatly from an accurate prediction of the function of future liver remnant (FLR) and assessment of risk of PHLF. Unfortunately, reliable and reproducible tests to predict function of remnant liver are currently not available in clinical practice. This results in either patients being turned down for potentially curative treatment or patients undergoing multiple interventions with additional risks including mortality when such interventions are not essential due to adequate function of remnant liver. Quantitative liver function tests such as indocyanine green (ICG), LiMAX (maximum liver function capacity) assess the liver as whole rather than the function of different parts of liver. Conventional cross sectional imaging like magnetic resonance imaging (MRI) and computerized tomography (CT) can provide an estimate of the volume of the future remnant, but this is inadequate in predicting the function in the presence of background liver disease like fibrosis, chemotherapy related injury, cirrhosis and cholestatsis. A potential solution is to combine whole liver function tests with FLR volume as determined on imaging ([10]; Y [11].; Yukihiro [12]), but this is based on an assumption that liver function is uniformly distributed across all liver segments. Hepatobiliary scintigraphy accounts for this factor [13–15], but is not widely available and is limited by inaccurate assessment of volume.

MRI-based liver function tests based on uptake of the liver-specific contrast agent Gadoxetate offer a promising approach for assessment of FLR function in clinical practice [16]. Apart from wide availability, a major advantage of Gadoxetate as compared to ICG and tracers used in hepatobiliary scintigraphy is the low extraction fraction even in healthy liver. With a fast dynamic acquisition and a pharmacokinetic analysis of the data (dynamic Gadoxetate-enhanced MRI or DGE-MRI), this enables a separate quantification of liver perfusion and hepatocellular function, as well as structural parameters such as the extracellular volume fraction [17]. Previous studies on Gadoxetate MRI for liver surgery planning use descriptive or approximate measures of Gadoxetate uptake and therefore do not fully exploit the potential offered by this extra quantitative information [18–20]. Moreover, previous work tends to focus on HCC, but inhomogeneous liver function can be relevant even in patients with liver metastases due to the effects of chemotherapy-induced liver injury and obesity-associated liver disease [21].

A pilot study with DGE-MRI in 29 patients with colorectal liver metastases (CRLM), using post-operative bilirubin as a surrogate for liver function, suggested that DGE-MRI biomarkers significantly improved the predictions of post-operative function compared to biochemical markers and volumetric assessment of the FLR [22]. A parameter of particular interest was the normalised Gadoxetate clearance (GC), a combination of perfusion and function that determines the rate at which the liver clears Gadoxetate from the blood pool. GC has units of mL/min/kg and measures the volume of plasma (mL) fully cleared of Gadoxetate per minute and per kg body weight. Potentially, the GC of the FLR (FLR-GC) can offer a clear and widely available cut-off value to determine if a surgery is safe to perform, irrespective of the underlying disease or the body size of the patient considered for surgery.

The primary aim of HEPARIM is to determine if DGE-MRI biomarkers of liver function and perfusion offer a significant improvement in the prediction of post-hepatectomy liver function. ICG clearance will be used as a reference measurement of post-operative liver function and correlated against DGE-MRI before surgery. As a secondary aim, DGE-MRI will be validated directly by performing ICG pre-operatively at the same time as the MRI and correlating the ICG clearance with whole-liver GC (WL-GC). A positive result on the primary aim can potentially improve the safety profile of hepatic surgery and open the door to extend the indication to wider groups of patients and avoid harmful interventions where they are not required.

## Methods/design

### Objectives and outcome measures

#### Primary objective

To determine if DGE-MRI can improve predictions of post-hepatectomy liver function. The primary outcome measure is the correlation between preoperative FLR-GC (mL/min/kg) and ICG R15 1 day after surgery (%).

#### Secondary objectives

1. To validate DGE-MRI based measurements of liver function against a gold-standard liver function test. The outcome measure is the correlation between preoperative WL-GC (mL/min/kg) and a simultaneously measured ICG R15 (%).
2. To determine the relationship between volumetric and functional liver growth after PVE/ALPPS. Outcome measure is the correlation between growth in FLR volume and growth in FLR-GC after the first stage.
3. To determine if preoperative DGE-MRI can improve predictions of PHLF (any grade) over and above routinely available data. HEPARIM will adopt the PHLF definition by the International Study Group of Liver Surgery (ISGLS).
4. To determine if preoperative DGE-MRI can improve predictions of 90-day post-operative outcome over and above routinely available data. (*Hepatectomy Risk Assessment With Functional Magnetic Resonance Imaging - Full Text View -* *ClinicalTrials.gov*, n.d.)

### Study design

#### Summary

HEPARIM is an observational cohort study that will aim to recruit 134 patients referred locally for a one- or two-stage liver resection of 2 segments or more. Participants will undergo a preoperative ICG liver function test and a DGE-MRI scan of the liver, and another ICG test at 1 day after surgery. Additional pre- and postoperative data will be collected from their medical records including demographics, routine imaging, biochemistry, pathology and radiology reports, any data collected in the 90-day follow-up visit. Participants who will undergo a staged resection with either PVE or ALLPS will have additional DCE-MRI and ICG tests before the first stage.

#### Study authorizations

The HEPARIM study has been approved by the North West - Liverpool Central Research Ethics Committee (IRAS project ID: IRAS 240787, REC reference: 19/NW/0139). The study sponsor is University of Leeds.

#### Setting

A single high-volume tertiary referral hepatobiliary surgery center in the United Kingdom (Leeds Teaching Hospitals Trust).

#### Timing

Recruitment was scheduled to occur over a two-year period which began on 1st July 2019. The timing has been significantly disrupted due to the ongoing COVID-19 pandemic, ongoing at the time of writing. Patients will be followed up for a three-month period post-operatively. Recruitment will end when the target number of patients have been recruited and have completed their imaging, surgery and follow up, or when the research team feel that continuing the study is either unsafe or unfeasible.

#### Data analysis

A univariable analysis will be performed comparing preoperative MR predictions of post-operative liver function against post-operative ICG-R15 (primary objective) and comparing preoperative MR measures of whole liver function against preoperative ICG-R15 (secondary objective). Second the correlations will be investigated in a multivariable analysis to determine if MRI adds value to existing preoperative biomarkers for predicting PHLF and 90-day outcome. Multivariate models will be derived for predicting both outcomes and it will then be tested whether this combined score is statistically significantly worse when DGE-MRI is removed from the prediction. In the PVE/ALPPS group, changes in volume will be correlated with changes in function of the FLR between pre PVE/pre-ALPPS and post-PVE/post-ALPPS.

### Study participants

#### Inclusion criteria

Adults over 18 years of age and under 80 years

- Referred to the hepatobiliary surgical service at St James’s Hospital
- Diagnosed with any hepatic tumour
- One of:
- Referred for liver resection of two or more Couinaud segments by multi-disciplinary team discussion at Saint James’s Hospital (Primary resection arm)
- Referred for PVE or ALPPS prior to likely major hepatectomy by multi-disciplinary team discussion at Saint James’s Hospital (PVE/ALPPS arm)

#### Exclusion criteria

- Unable to consent independently
- Previous liver resection
- Private patients
- Concurrent malignancy unrelated to liver tumour
- Chronic renal failure (eGFR < 30 mL/min)
- Possible pregnancy
- Other contraindications to DGE-MRI, including:
- Cochlear Implant
- Aneurysm Clips
- Neurological stimulator
- Implanted cardiac devices (ICD, PPM, loop recorders, or any others)
- Metal heart valve
- History of metal foreign bodies in orbits
- Other implanted metal device which prevents MR imaging
- Known allergy to Gadolinium contrast
- Claustrophobia
- Weight exceeding 250 kg
- Maximal diameter exceeding 55 cm
- Other contraindications to ICG, including:
- Known allergy to Indocyanine green
- Known allergy to sodium iodine
- Known reaction or allergy to iodine
- Previous diagnosis of a thyroid problem
- Breastfeeding

(*Hepatectomy Risk Assessment With Functional Magnetic Resonance Imaging - Full Text View -* *ClinicalTrials.gov*, n.d.)*Hepatectomy Risk Assessment With Functional Magnetic Resonance Imaging - Full Text View -* *ClinicalTrials.gov*. (n.d.). Retrieved September 30, 2021, from https://clinicaltrials.gov/ct2/show/NCT04705194

### Study procedures (resection arm)

**Fig. 1:**
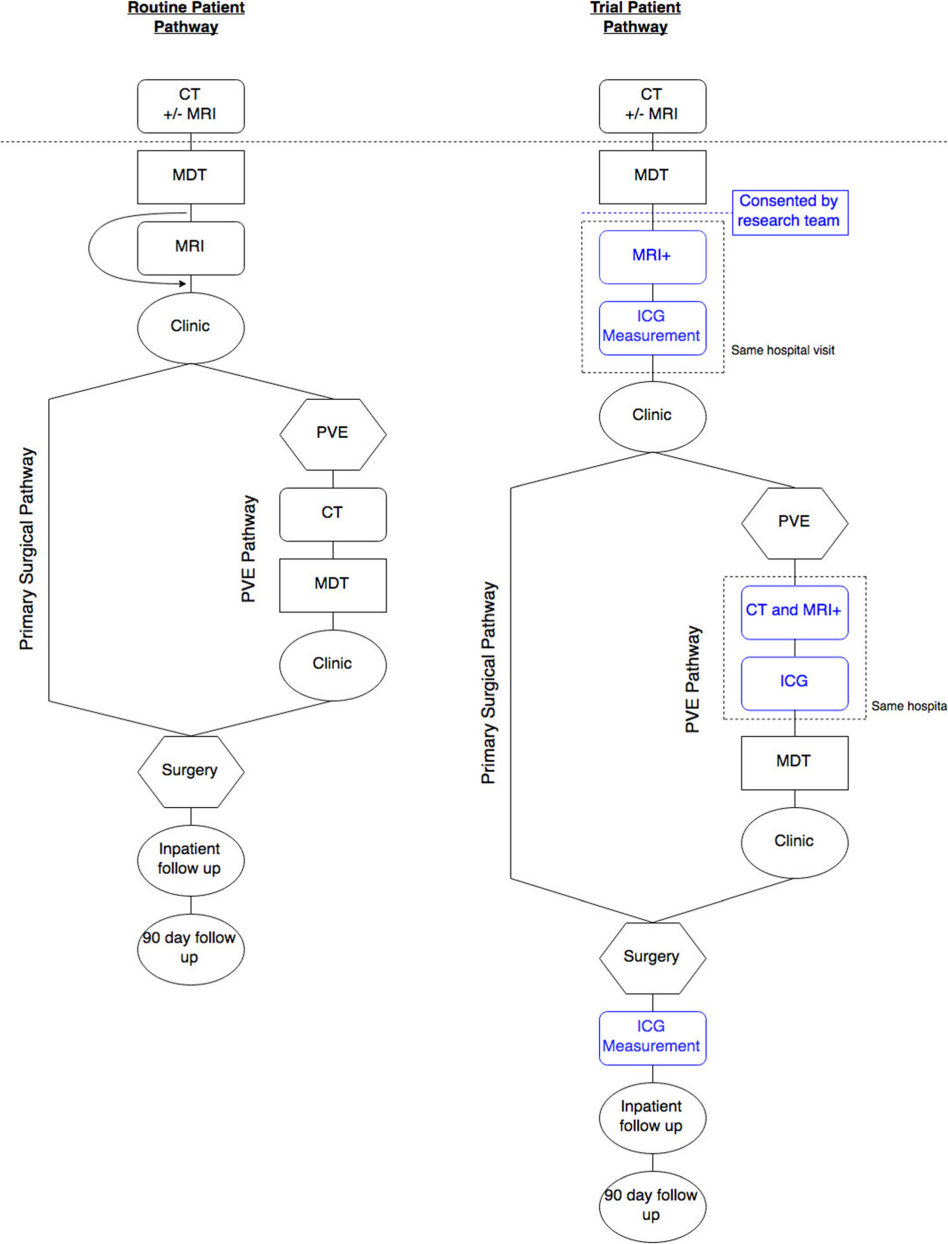
Pathway demonstrating patients route through the trial in both the primary surgical and portal vein embolisation arms. Items in blue are unique to trial patients

HEPARIM will involve only a minimal deviation from the routine patient pathway (Fig. 1). In about 3/4 of participants the only investigations added for research purposes are the ICG measurements. The rest will not require a new MRI for surgery planning, and an additional pre-surgery MRI would therefore be performed for research purposes only. In the small number of PVE/ALPPS patients, the additional MRI is performed only for research purposes – see section on study procedures for the PVE/ALPSS arm.

#### Recruitment

Patients considered for major liver resection will be identified at the Hepatobiliary multi-disciplinary team (MDT) meeting at St James University Hospital Leeds and contacted by phone. If they agree to finding out more about the study then an information pack will be posted to them, including a letter of invitation, the patient information sheet and an example consent form. Patients will have at least 3 days to consider this information, and after that they will be contacted by the member of their clinical care team to schedule the baseline visit.

#### Baseline visit

*Consenting*: Formal written consent will be obtained on the day of the visit. After consenting the participant will be assigned an anonymous study ID. If they don’t consent to participation, then only the MRI scans requested by the MDT will be collected and stored in their medical records, but no research data will be collected.

##### Questionnaires

An initial dataset will be captured incorporating the participant’s medical history and up-to-date investigations. This will be obtained through a combination of interview, review of paper notes and review of the electronic medical record. An MRI safety screening form will be administered. If a contraindication to MRI is found at this point, the visit will be cancelled, and the participant will be excluded from the study. An ICG safety questionnaire will be used to exclude participants with contraindications to ICG use. Participants excluded from ICG testing at this point will not be excluded from the study as other measures of liver function will still be available for analysis of secondary outcomes.

##### ICG measurement

Participants will be cannulated and ICG will be administered intravenously as a bolus dose of 0.5 mg/kg through the cannula. The ICG will be reconstituted with 5 ml of water for injection for every 25 mg of ICG, leading to a dilution of 5 mg ICG per 1 ml. Serial percutaneous ICG measurements will then be taken using an ICG clearance meter using a finger clip device (LiMON; Maquet, Germany). Participants will lie in the supine position and calibration will be performed before testing starts. In order to derive absolute ICG concentrations, we will also take two separate blood samples of 2.5 mL each, both after ICG injection (pre-operative). The samples will be processed and stored in a dedicated facility in the Leeds Institute for Cardiovascular and Metabolic Medicine (LICAMM) and analysed as a batch at the end of the study.

##### MRI scanning

The MRI will be performed straight after ICG measurement in 3 T Siemens (Erlangen, Germany) Magnetom Prisma scanner by radiographers of the department. The scan will last max 1.5 h and will include the injection of a standard dose of MRI contrast agent Gadoxetate 0.1 ml/kg (Primovist, Bayer AG). The same cannula will be used to administer he MRI contrast as for the ICG test. Other scans that will be taken in the same session include precontrast T1- and T2-weighted anatomical imaging, T2*-mapping, T1-mapping, proton-density fat fraction and post-contrast T1-weighted anatomical imaging.

#### Routine clinic visit

After completion of their MRI some participants will require re-discussion in the MDT and all participants will attend for a preoperative clinic appointment (see Fig. 1). A proportion of participants will not progress to surgery due to clinician or participant choice; these participants will not be followed up further. But the data that has already been collected will be used for addressing the secondary objective.

#### Intraoperative measurements

Intraoperative measurements of the resected liver segments will be taken, including weight and volume (measured using fluid displacement). The weight measurement is a routine procedure, but the volume measurement will be done for research purposes only.

#### In-patient follow-up

Once the participant’s surgery is complete, data obtained as part of the participant’s standard intra and post-operative care will be collected. This includes any blood tests during admission, the characteristics of the operation, complications and length of stay. Pathology results will be collected once reported. The ICG test with the finger-clip device will be repeated postoperatively at bedside to determine a reference value for postoperative liver function. This will be performed on day 1 post-surgery if feasible. A range of biochemical measures will be used to calculate postoperative liver function using the post-operative data set.

#### 90-day follow-up

After discharge all participants who underwent surgery attend the hospital for a routine post-operative clinic appointment after 90 days. Data from this appointment will be collected by the clinical research fellow, including complications, re-admissions and mortality.

### Study procedures (PVE/ALPPS arm)

Participants requiring PVE/ALPPS will be recruited in the same way as participants in the resection arm. They will attend the same baseline visit and the routine clinic visit. Participants may decline the PVE/ALPPS at this point, or the clinician may decide it is inappropriate at the clinic appointment. If this occurs and the participant proceeds to surgery, they will remain in the study. If they do not proceed to surgery their data will be used to address the secondary objectives.

A participant who does proceed to PVE/ALPPS will attend the hospital for a routine CT scan after the first stage, and a second research visit will be scheduled at that point. This visit will proceed in the same manner as the baseline visit. They will be re-discussed in the MDT and come for a preoperative clinic appointment. Participants may be deemed ineligible for surgery, or make the decision not to proceed with surgery, in which case their data already collected can feed into the secondary objectives. If they do proceed to surgery, the same intra-operative and post-operative data will be taken as for the resection arm.

### Management of data and samples

#### Patient-identifiable data

All physical paperwork with identifiable data will be stored in a secure location in St James’s Hospital. Electronic records with identifiable data will be encrypted, password protected and stored on the hospital’s secure servers. This includes a single key file containing the link between patient ID and anonymous study ID. Identifiable MRI data will also be stored in the secure Picture Archiving and Communication System (PACS) of the radiology department. Identifiable data will not be accessed by researchers outside of the clinical care team and are only retained for quality control, backup, and to follow-up on any incidental findings.

#### Pseudo-anonymised study data

Data collected for research purposes will be recorded using the participant’s anonymous study ID, in a single file per patient (See Clinical Data points supplementary file 1). Data will subsequently be transferred to an excel sheet for statistical analysis. Paper forms will be stored in a secure location in St James University Hospital. Electronic data will be stored in a participant study record in a secure database on an NHS workstation, and in password protected network drives set up and maintained by University IT. MRI images will be transferred anonymously from the scanner to the university’s database, where they will be analyzed by a research fellow to extract the imaging biomarkers. The original MRI data will be retained the university’s database to enable future secondary analyses.

#### Blinding and clinical review of study data

All data collected for the study, except calculated MRI biomarkers and ICG measurements, are acquired as part of the routine workup and will therefore be available to the clinical care team through the participant’s medical records. ICG liver function measurements will be made available to the clinical care team to inform management as appropriate. MRI scans will be reviewed by the radiology department and any unusual findings will be flagged up to the clinical care team. The research fellows working on MRI data processing and statistical analysis will be blinded to the clinical history of the participant and other function tests.

#### Long-term data storage

Study documents with identifiable data will be retained for 10 years in line with the MRC Good Research Practice guidelines (Medical Research Council 2012) and then destroyed using the hospital’s confidential waste management service. During this ten-year period data will be stored securely on the hospital’s premises and secure electronic systems. Access to the data during this period will only be available with the consent of one of the study’s clinical supervisors.

All excel sheets with anonymised study data will be transferred to a long-term data repository where they will be maintained for a minimum of 25 years. Source data for MRI and ICG will be retained indefinitely in a dedicated imaging data management system hosted by the University to enable secondary research, educational use, and data sharing.

#### Withdrawal of participants

If a participant decides to withdraw their consent to inclusion in the study at any point then no further data regarding their care will be collected. Should the participant wish, data collected up until withdrawal of consent will be destroyed. Participants who lose their capacity to consent will be treated in the same manner as if they had withdrawn consent. Previously collected data will not be destroyed. If a participant is unable to complete their MRI study for any reason they will automatically be withdrawn from the study. Data collected until the point of withdrawal will be retained, unless the participant requests they are deleted,

#### Blood samples

Blood samples taken after ICG injection at the baseline visit will be processed in the LICAMM laboratories. Whole blood and blood plasma will be stored in a secure sample storage facility within LICAMM with access limited to LICAMM staff. Samples will be stored in a freezer and will only be accessed by members of the research team. ICG concentrations will be measured in batch after finalizing recruitment. The remaining samples will be stored for a maximum of 5 years to measure metabolomics data for secondary research. Precise use will be based on future research priorities. After 5 years any remaining samples will be destroyed according to standard LICAMM procedure for sample disposal.

### Sample size considerations

#### Audit

Sample size calculation was informed by an audit of the preceding year. Between October 2016 and October 2017, a total of 165 patients who met the inclusion criteria underwent surgery at St James’s hospital. 15% of these patients underwent PVE. Patients undergoing major liver resection had a rate of PHLF equal to 9%, although the rate of those requiring medical intervention as a result of their PHLF was only 3.6%. Of the eligible patients, 67% had an MRI in Leeds after the MDT, and for a further 7% the MDT requested a local MRI.

For practical and funding reasons the maximum duration of patient recruitment in HEPARIM was set at 2 years. Based on experience in a pilot study [22], a 50% recruitment rate was assumed in patients that do not require an additional MRI visit for research purposes. A 25% recruitment rate was assumed in primary surgery patients that need an additional MRI, and in PVE/ALPPS patients.

With these assumptions the maximum number of participants over the 2 years is 134, including 122 into the primary surgery arm and 12 into the PVE/ALPPS arm. The pilot study also demonstrated that 18% of patients recruited into the study ultimately did not undergo surgery. Hence only 82% of the 122 recruited patients in the surgery arm will feed into the primary objective, or a total of 112 including those in the PVE/ALPPS arm.

#### Power calculation

The power calculation was informed by pilot data in 29 cases, without ICG measurement [22]. The following assumptions were made: (1) patient population values of FLR-GC follow a log-normal distribution; (2) ICG-C = 10 x FLR-GC + e, with an error variance that reflects uncertainty in this relationship. The factor 10 encodes the fact that ICG extraction fractions are an order of magnitude higher than for Gadoxetate; (3) error variance is proportional to true FLR-GC, which invokes heteroscedasticity, as observed for the pilot data.

The impact of study size was assessed by simulating a hypothetical population of 1 million patients and evaluating model robustness to estimate the model beta coefficient of FLR-GC in relation to ICG-C. The relationship between FLR-GC and ICG-C values is modelled for different error variances ranging from very low precision (4 times the variance of FLR-GC) to good precision (one quarter the variance of FLR-GC). 10 k samples of 120 patients are drawn with replacement for each of the three error variances, generating empirical distributions of the parameters.

**Fig. 2:**
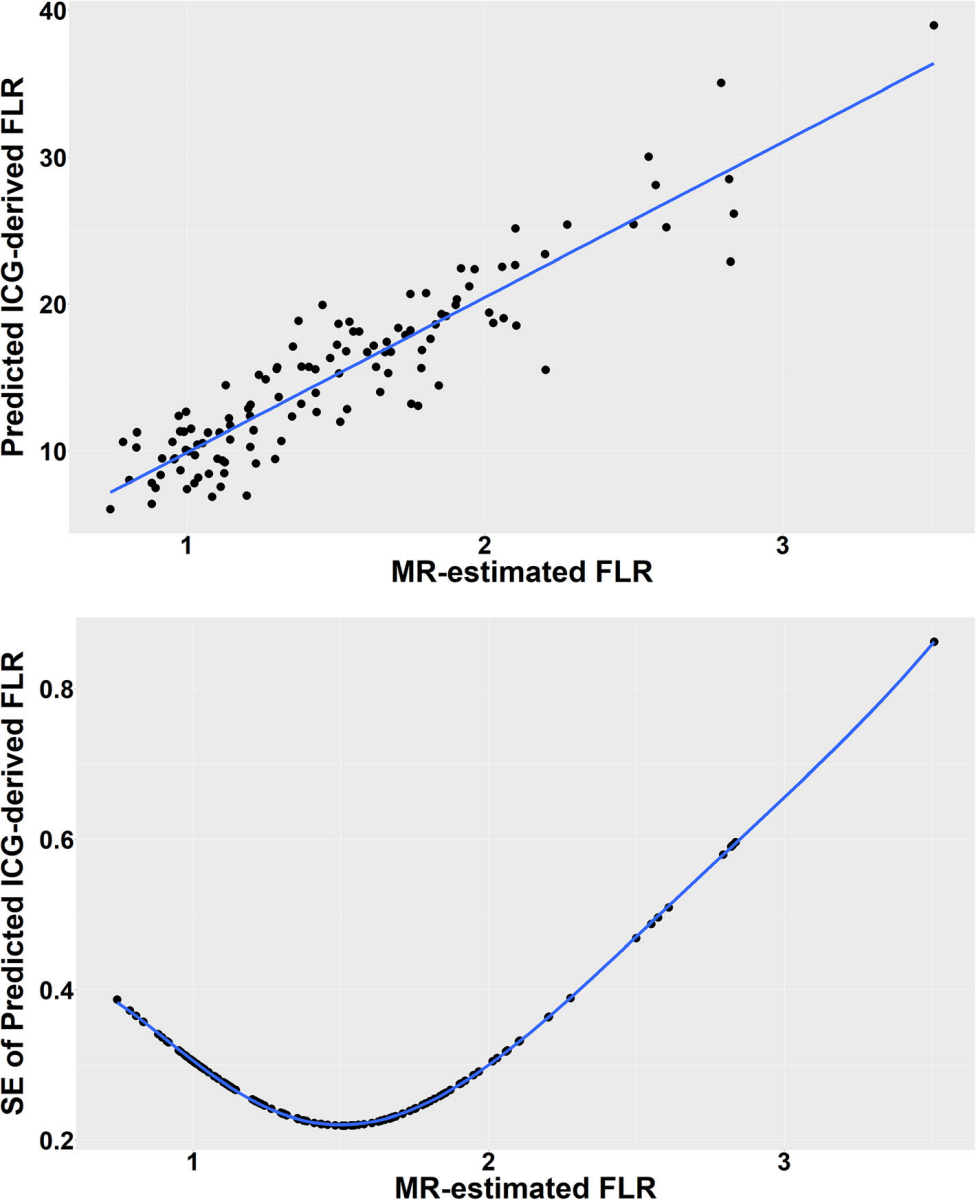
Predicted ICG-C (top) and error in the predicted ICG-C (bottom) for the lowest precision simulated

Figure 2 shows an example with error variance reflecting low precision. A plot of the model standard errors is also shown and reveals how optimal precision is around the largest cluster of data points towards the lower range of FLR-GC values (1.3 mL/min/kg). At this lowest precision, the 95% confidence interval for the model beta coefficient ranges from 9.12 to 10.88 with a median of 10.01 – very close to the exact value of 10. The half-width of the 95% CI is 8.8% of the exact value, showing that the sample size is sufficient to obtain reliable beta coefficients even at the poorest precision simulated.

## Discussion

Despite recent changes in surgical practice with increased appetite towards parenchymal preserving surgery for superficial liver tumours, patients with multiple, deep or perivascular lesions still require major resectional surgery and are not amenable to simple metastasectomy. For these patients PHLF remains a major concern, the risk of which can potentially be reduced with a reliable and widely available prediction of post-operative function. Such a test could also help guide patient selection for PVE, two stage surgery and ALLPS and determine which patients do in fact need portal embolisation and which patients can safely proceed straight to surgery. Once selected for PVE, two stage surgery or ALPPS, a partial liver function test may improve decisions on the optimal time interval between vascular intervention and surgery. This can potentially reduce the risk of further tumour invasion or growth in the waiting period between the two surgical stages.

Currently available liver function tests are limited either by their inability to assess regional segmental liver function directly or are not widely available. The HEPARIM trial will examine the ability of a novel MRI-based technique to accurately measure FLR function and predict PHLF using FLR-GC as an index of direct regional hepatocyte function. Being an MRI modality, it can be embedded in the current pre-operative diagnostic algorithm without the need for additional procedures in most patients and in a cost-effective manner. Assuming a positive outcome, HEPARIM will produce strong hypotheses that can inform improved management strategies and future clinical trials to test their safety and efficacy. Potentially, this could include extending the indication for surgery in a subgroup of liver cancer patients that are currently considered inoperable due to the high risk of PHLF, but who have a poor prognosis without surgery.

## Supplementary Information

**Additional file 1.**

## Data Availability

The data sharing policy for HEPARIM is specified in the Data Management Plan of the funding application and in line with MRC requirements and guidelines. All data will be made available for secondary research by other internal or external investigators, and for educational or commercial purposes. Suitability for sharing: All anonymised data are suitable for sharing. One reason for sharing is to allow independent verification by other researchers, or to develop novel data processing and modeling methods that produce novel biomarkers or more accurate values. The data will also be useful for validating implementations in other centres, or as part of a future quality assurance process. Discovery by potential users of the research data: A project website will be set up which will contain basic information about the project, investigators, results and news, access to software and training materials, and also examples of the data and modalities for data sharing. Governance of access: The decision process for supplying the data will follow the data sharing request workflow as specified on p11 in the MRC policy and guidance on Sharing of Research data. For this study, requests will be approved following model 2. The study team’s exclusive use of the data: The study team’s exclusive use of the data will be restricted to the duration of the study. Restrictions or delays to sharing: The patient consent form contains a section explaining the use of the data for the research project and future exploitation by third parties, and the conditions under which they will be released. Patients will only be enrolled in the study if they consent to these modalities. There are no restrictions or delays to sharing expected. Regulation of responsibilities of users: Users will need to sign a data sharing agreement. This will lay out user responsibilities such as checking for internal review board approval, making no attempts to establish the identity of the subjects, adhering to redistribution rights and licensing, acknowledging the funding body and authors in future publications.

## Abbreviations

AEs: All adverse events
ALPPS: Associating liver partition and portal vein ligation for staged hepatectomy
CCA: Cholangiocarcinoma
CRC: Colorectal cancer
CT: Computerized tomography
DGE- MRI: Dynamic gadoextate enhanced magnetic resonance imaging
FLR: Future liver remnant
GC: Gadoxetate clearance
HPB: Hepatobiliary
HCC: Hepatocellular carcinoma
ICD: Implantable cardiac device
ICG: Indocyanine green
MDT: Multi-disciplinary team
MRI: Magnetic resonance imaging
PHLF: Post hepatectomy liver failure
PPM: Permanent pacemaker
PVE: Portal vein embolisation
R15: Retention rate at 15 min
SAEs: Serious adverse events
WH: Whole liver
