# Supplementary material for: Hepatectomy risk assessment with functional magnetic resonance imaging (HEPARIM)
Source: BMC Cancer. 2021 Oct 23;21:1139. doi: 10.1186/s12885-021-08830-4 (PMC8541801; doi:10.1186/s12885-021-08830-4)
Supplement: Supplementary file 1 — Additional file 1. [file 12885_2021_8830_MOESM1_ESM.docx]

Clinical Data Points

HEPARIM Trial

## Version 1.1

## 02/03/2021

# Authors:

Mr Magdy Attia

Dr Ashley Guthrie

Mr Matthew Roche

Dr Ian Rowe

Steven Sourbron

Mr Dhakshina Vijayanand

# Induction Visit - Data Elements

| **Data Field** | **Data Elements** | **Comment** |
| --- | --- | --- |
| **Identification** | | |
| Patient Initials | Text |  |
| Date of visit | Date / / |  |
| Gender | Male / Female |  |
| Age | Number |  |
| Nationality | Text |  |
| Racial Background | White European  Asian  Black or African American  American Indian  Other |  |
| **Inclusion Criteria** | | |
| Diagnosis | CRLM / HCC / CholCa |  |
| Referred for | Resection / PVE |  |
| Require additional MRI | Yes / No |  |
| **Exclusion Criteria** | | |
| Capacity to consent | Yes / No |  |
| Previous liver resection | Yes / No |  |
| Private patient | Yes / No |  |
| Pregnancy | Yes / No / NA |  |
| Other malignancy | Yes / No |  |
| Included in other study | Yes / No |  |
| Allergy to gadoxetate | Yes / No |  |
| History of chronic renal failure | Yes / No |  |
| **Eligibility** | | |
| Eligible | Yes / No |  |
| **Current Diagnosis** | | |
| Diagnosis Date | Date |  |
| Diagnosis Location | Text |  |
| **Past Medical History** | | |
| Past Medical History | Text |  |
| Past Surgical History | Text |  |
| Previous Chemotherapy | Text |  |
| Previous Radiotherapy | Text |  |
| **Medications** | | |
| Current Medications | Text |  |
| Allergies | Text |  |
| **Social History** | | |
| Occupation | Text |  |
| Smoking History | Current / Ex-smoker |  |
| Alcohol History | Text |  |
| Illicit Drug History | Text |  |
| Performance Status | 0 / 1 / 2 / 3 / 4 / 5 |  |
| **Biometrics** | | |
| Height | Text |  |
| Weight | Text |  |
| Waist Size | Text |  |
| **Referral Information** | | |
| Referral Source | Text |  |
| Referral Date | Date |  |
| MDT Date | Date |  |
| **Prior Investigations** | | |
| CT Report | Text |  |
| CT Date | Date |  |
| MRI Report | Text |  |
| MRI Date | Date |  |
| Latest Blood Results | Hb:  MCV:  WCC:  Neu:  Lymph:  Na:  K:  Ur:  Cr:  eGFR:  Bil:  ALT:  ALP:  Alb:  INR: |  |
| Latest Blood Results Date | Date |  |
| Other Significant Investigation | Text |  |

# ICG – Data Elements

| Date of investigation | Date |  |
| --- | --- | --- |
| **ICG Testing (fingerclip)** | | |
| ICG Testing Performed | Yes / No |  |
| Time of ICG testing | Time |  |
| Fasted prior to attending | Yes / No |  |
| ICG Safety Questionnaire Completed | Yes / No |  |
| ICG Dose Given | Text |  |
| Cannula Size | Text |  |
| Adverse Reaction | Yes / No |  |
| R15 Value | Text |  |
| PDR Value | Text |  |
| **ICG Testing (blood analysis)** | | |
| Sample 1 time taken | (minutes after injection) |  |
| Sample 1 ICG concentration |  |  |
| Sample 2 time taken | (minutes after injection) |  |
| Sample 2 ICG concentration |  |  |

# MRI – Data Elements

| Date of Investigation | Date |  |
| --- | --- | --- |
| **MRI** | | |
| MRI Performed | Yes / No |  |
| MRI Safety Questionnaire Completed | Yes / No |  |
| Time of MRI | Time |  |
| Gadoxetate Dose | Text |  |
| MRI Completed | Yes / No |  |

| **Imaging Biomarker Name** | **Unit** | **Regions** |
| --- | --- | --- |
| Volume | ml | Liver, FLR, segment 1-8 |
| Relative Volume | % | FLR, segment 1-8 |
| Venous perfusion | mL/min/100mL | Liver, FLR, segment 1-8 |
| Arterial perfusion | mL/min/100mL | Liver, FLR, segment 1-8 |
| Intracellular uptake rate | mL/min/100mL | Liver, FLR, segment 1-8 |
| Biliary excretion rate | mL/min/100mL | Liver, FLR, segment 1-8 |
| Extracellular Volume | mL/100mL | Liver, FLR, segment 1-8 |
| Venous blood flow | mL/min | Liver, FLR, segment 1-8 |
| Arterial blood flow | mL/min | Liver, FLR, segment 1-8 |
| Uptake function | mL/min | Liver, FLR, segment 1-8 |
| Clearance function | mL/min | Liver, FLR, segment 1-8 |
| Relative Uptake function | % | FLR, segment 1-8 |
| Normalized clearance | mL/min/kg | Liver, FLR, segment 1-8 |

# Intra-operative – Data Elements

| Date of operation | Date |  |
| --- | --- | --- |
| **Administrative** | | |
| Location of surgery | Text |  |
| Time of surgery |  |  |
| Primary Surgeon | Text |  |
| Assisting Surgeons(s) | Text |  |
| Length of surgery | Text |  |
| **Operative Details** | | |
| Actual segments resected | Text |  |
| Estimated blood loss | Text |  |
| Intra-operative blood transfusion | Yes / No |  |
| Intra-operative complication | Text |  |
| **Resection Details** | | |
| Sample weight | Text |  |
| Sample volume | Text |  |

# Resection summary

| Segment | Actual volume resected (mL) | Notes |
| --- | --- | --- |
| 1 |  |  |
| 2 |  |  |
| 3 |  |  |
| 4 |  |  |
| 5 |  |  |
| 6 |  |  |
| 7 |  |  |
| 8 |  |  |

# Post-operative – Inpatient – Data Elements

| **Post-op Day 1** | | |
| --- | --- | --- |
| Location | ITU / HDU / Ward |  |
| Sedation | Yes / No |  |
| Ventilation | Yes / No |  |
| Oxygen Therapy | Yes / No |  |
| Inotrope Support | Yes / No |  |
| Renal Support Therapy | Yes / No |  |
| Bloods | Hb:  MCV:  WCC:  Neu:  Lymph:  Na:  K:  Ur:  Cr:  eGFR:  Bil:  ALT:  ALP:  Alb:  INR: |  |
| **Post-op Day 2** | | |
| Location | ITU / HDU / Ward |  |
| Sedation | Yes / No |  |
| Ventilation | Yes / No |  |
| Oxygen Therapy | Yes / No |  |
| Inotrope Support | Yes / No |  |
| Renal Support Therapy | Yes / No |  |
| Bloods | Hb:  MCV:  WCC:  Neu:  Lymph:  Na:  K:  Ur:  Cr:  eGFR:  Bil:  ALT:  ALP:  Alb:  INR: |  |
| **Post-op Day 3** | | |
| Location | ITU / HDU / Ward |  |
| Sedation | Yes / No |  |
| Ventilation | Yes / No |  |
| Oxygen Therapy | Yes / No |  |
| Inotrope Support | Yes / No |  |
| Renal Support Therapy | Yes / No |  |
| Bloods | Hb:  MCV:  WCC:  Neu:  Lymph:  Na:  K:  Ur:  Cr:  eGFR:  Bil:  ALT:  ALP:  Alb:  INR: |  |
| **Post-op Day 5** | | |
| Location | ITU / HDU / Ward |  |
| Sedation | Yes / No |  |
| Ventilation | Yes / No |  |
| Oxygen Therapy | Yes / No |  |
| Inotrope Support | Yes / No |  |
| Renal Support Therapy | Yes / No |  |
| Bloods | Hb:  MCV:  WCC:  Neu:  Lymph:  Na:  K:  Ur:  Cr:  eGFR:  Bil:  ALT:  ALP:  Alb:  INR: |  |
| **Post-op Day 10** | | |
| Location | ITU / HDU / Ward |  |
| Sedation | Yes / No |  |
| Ventilation | Yes / No |  |
| Oxygen Therapy | Yes / No |  |
| Inotrope Support | Yes / No |  |
| Renal Support Therapy | Yes / No |  |
| Bloods | Hb:  MCV:  WCC:  Neu:  Lymph:  Na:  K:  Ur:  Cr:  eGFR:  Bil:  ALT:  ALP:  Alb:  INR: |  |
| **Post op ICG Testing** | | |
| ICG Testing Performed | Yes / No |  |
| Time of ICG testing | Time |  |
| Fasted prior to test | Yes / No |  |
| ICG Safety Questionnaire Completed | Yes / No |  |
| ICG Dose Given | Text |  |
| Cannula Size | Text |  |
| Adverse Reaction | Yes / No |  |
| R15 Value | Text |  |
| PDR Value | Text |  |
| **Post-operative Complications** | | |
| List of complications | Text |  |
| Highest Clavien-Dindo grade | Text |  |
| **Return to theatre** | | |
| Return to theatre | Yes / No |  |
| Reason for return | Text |  |
| Outcome | Text |  |
| **Discharge** | | |
| Discharge Date | Date |  |
| Discharge To | Home / Other |  |
| Discharge Summary Text | Text |  |
| **Death** | | |
| Patient deceased | Yes / No |  |
| Date of death | Date |  |
| Cause of death | Text |  |

# Post-operative – 90-day follow up – Data elements

| Date of follow-up | Date |  |
| --- | --- | --- |
| **Administrative** | | |
| Location | Text |  |
| Time | Time |  |
| **Readmission** | | |
| Readmission since discharge | Yes / No |  |
| Reason for readmission | Text |  |
| Readmission Outcome | Discharge / Death |  |
| **Post-operative Complications** | | |
| List of complications | Text |  |
| Highest Clavien-Dindo grade | Text |  |
| **Return to theatre** | | |
| Return to theatre | Yes / No |  |
| Reason for return | Text |  |
| Outcome | Text |  |
| **Death** | | |
| Patient deceased | Yes / No |  |
| Date of death | Date |  |
| Cause of death | Text |  |

# Supplementary material with all case notes (anonymised)

- Preoperative MRI radiology report
- Pathology report
- Surgical notes
